# Metabolic Potential of the Superphylum *Patescibacteria* Reconstructed from Activated Sludge Samples from a Municipal Wastewater Treatment Plant

**DOI:** 10.1264/jsme2.ME22012

**Published:** 2022-06-28

**Authors:** Naoki Fujii, Kyohei Kuroda, Takashi Narihiro, Yoshiteru Aoi, Noriatsu Ozaki, Akiyoshi Ohashi, Tomonori Kindaichi

**Affiliations:** 1 Department of Civil and Environmental Engineering, Graduate School of Advanced Science and Engineering, Hiroshima University, 1–4–1, Kagamiyama, Higashihiroshima, Hiroshima 739–8527, Japan; 2 Bioproduction Research Institute, National Institute of Advanced Industrial Science and Technology (AIST), 2–17–2–1 Tsukisamu-Higashi, Toyohira-ku, Sapporo, Hokkaido 062–8517, Japan; 3 Program of Biotechnology, Graduate School of Integrated Sciences for Life, Hiroshima University, 1–3–1, Kagamiyama, Higashihiroshima, Hiroshima 739–8530, Japan

**Keywords:** *Patescibacteria*, candidate phyla radiation (CPR), activated sludge, metagenomic ana­lysis, *Chitinophagales*

## Abstract

*Patescibacteria* are widely distributed in various environments and often detected in activated sludge. However, limited information is currently available on their phylogeny, morphology, and ecophysiological role in activated sludge or interactions with other microorganisms. In the present study, we identified microorganisms that interacted with *Patescibacteria* in activated sludge via a correlation ana­lysis using the 16S rRNA gene, and predicted the metabolic potential of *Patescibacteria* using a metagenomic ana­lysis. The metagenome-assembled genomes of *Patescibacteria* consisted of three *Saccharimonadia*, three *Parcubacteria*, and one *Gracilibacteria*, and showed a strong positive correlation of relative abundance with *Chitinophagales*. Metabolic predictions from ten recovered patescibacterial and five *Chitinophagales* metagenome-assembled genomes supported mutualistic interactions between a member of *Saccharimonadia* and *Chitinophagales* via N-acetylglucosamine, between a member of *Parcubacteria* and *Chitinophagales* via nitrogen compounds related to denitrification, and between *Gracilibacteria* and *Chitinophagales* via phospholipids in activated sludge. The present results indicate that various interactions between *Patescibacteria* and *Chitinophagales* are important for the survival of *Patescibacteria* in activated sludge ecosystems.

The phylogeny and physiology of microorganisms in activated sludge for wastewater treatment remain unclear due to their complexity and variations even though the activated sludge process has been used in wastewater treatment globally for more than 100 years. The structure of a microbial community depends on the climate, location, environment, and process configuration of the wastewater treatment plant (Zhang *et al.*, 2012). Recent studies reported that communities appeared to be stable at the genus and substrate specificity levels (Nielsen *et al.*, 2010; Kindaichi *et al.*, 2013). Most microorganisms, including those in nature and in engineered systems, cannot be cultured in laboratories and are called “microbial dark matter” (Rinke *et al.*, 2013). In this context, molecular biological methods, such as a 16S rRNA gene ana­lysis and metagenomic ana­lysis, have been widely used to predict the metabolic functions of uncultured bacteria. Large metagenomic data obtained from samples in various environments revealed the existence of a large group of bacteria, called *Patescibacteria* or candidate phyla radiation (CPR) (Rinke *et al.*, 2013; Brown *et al.*, 2015). The *Patescibacteria* or CPR (hereafter called *Patescibacteria*) group includes 35 phyla, accounts for 15–50% of all bacterial phyla, and has been reported to exist in various environments (Brown *et al.*, 2015; Hug *et al.*, 2016; Takebe *et al.*, 2020). To date, only a few of its members (*i.e.*, phyla *Saccharimonadia* and *Gracilibacteria*) have been cultured (Soro *et al.*, 2014; He *et al.*, 2015; Ibrahim *et al.*, 2021; Yakimov *et al.*, 2021). These bacteria are parasitic on other bacteria for sustenance; however, since most of them cannot be cultured, the mechanisms underlying their existence are unclear. *Patescibacteria* are commonly characterized by a small genome size (approximately 1.0‍ ‍Mbp) (Lemos *et al.*, 2020; Nakai, 2020), limited metabolic potential, and fermentation-based metabolism (Wrighton *et al.*, 2012, 2014; Albertsen *et al.*, 2013; Lemos *et al.*, 2020). However, the physiology and phylogeny of *Patescibacteria* have not yet been elucidated in detail, except for some cultures in the phyla *Saccharimonadia* and *Gracilibacteria* (Soro *et al.*, 2014; He *et al.*, 2015; Moreira *et al.*, 2021; Yakimov *et al.*, 2021).

Activated sludge is an environment in which *Patescibacteria* are frequently detected. Among them, *Saccharimonadia*, *Parcubacteria*, and *Gracilibacteria* are the major phyla (Albertsen *et al.*, 2013; Kindaichi *et al.*, 2016; Singleton *et al.*, 2021). A moderate constituent of activated sludge is *Saccharimonadia*, a well-described *Patescibacteria* (Mielczarek *et al.*, 2012; Albertsen *et al.*, 2013; Kindaichi *et al.*, 2016). Based on the 16S rRNA gene classification, *Saccharimonadia* are primarily classified into three subdivisions, with members having a filamentous morphology belonging to subdivision 1, and members with a coccus or rod morphology belonging to subdivisions 2 and 3 (Hugenholtz *et al.*, 2001). A complete genome belonging to subdivision 3 was reconstructed from activated sludge samples and the data obtained showed that *Saccharimonadia* are obligate fermentative metabolic bacteria that use heterolactic fermentation pathways (Albertsen *et al.*, 2013). In addition, filamentous *Saccharimonadia* were detected in activated sludge from wastewater treatment plants, and the characteristics of substrate utilization elucidated by microautoradiography combined with fluorescence *in situ* hybridization (FISH) revealed more diverse carbon metabolism, including the utilization of oleic acid and amino acids, which was not predicted from the available genome (Kindaichi *et al.*, 2016). *Parcubacteria* also belong to *Patescibacteria* and are found in activated sludge (Zhang *et al.*, 2012). *Parcubacteria* have a diverse distribution within the phylum, with most members being frequently found in anaerobic environments, such as groundwater. *Parcubacteria* are considered to be involved in hydrogen production, sulfur reduction, and nitrite reduction (Wrighton *et al.*, 2012; 2014; Rinke *et al.*, 2013; Danczak *et al.*, 2017). Additionally, a syntrophic relationship with other bacteria has been suggested as a putative benzene degrader in anaerobic environments (Phan *et al.*, 2021). However, some members of *Parcubacteria* harbor genes that are capable of using O_2_ as a terminal electron acceptor (Nelson and Stegen, 2015). *Gracilibacteria* include three lineages and belong to *Patescibacteria*. Hanke *et al.* predicted that the terminal codon UGA encodes glycine in *Gracilibacteria* (Hanke *et al.*, 2014). These bacteria have poor metabolic potential (Sieber *et al.*, 2019), and some strains were reported to be parasitic on their hosts (Moreira *et al.*, 2021; Yakimov *et al.*, 2021).

Although many high-quality genomes related to *Patescibacteria* have been obtained from various environments, their detailed phylogeny, morphology, and ecophysiological role in activated sludge remain largely unknown. To clarify the phylogenetic and physiological diversities of *Patescibacteria* in activated sludge, obtaining high-quality genomes of *Patescibacteria* is necessary for further investigations in terms of visualization, *in situ* substrate utilization, and isolation. The purpose of the present study was to predict the metabolic potential of *Patescibacteria* in activated sludge and estimate their physiological role in activated sludge. A metagenomic approach using three activated sludge samples from a municipal wastewater treatment plant recovered 10 metagenome-assembled genomes (MAGs) related to *Saccharimonadia*, *Parcubacteria*, *and Gracilibacteria* within the superphylum *Patescibacteria*.

## Materials and Methods

### Sample collection

Four activated sludge samples were collected from aeration tanks in a wastewater treatment plant in Higashihiroshima city, which had previously been sampled (Kindaichi *et al.*, 2016; Table S1) in February 2019 (designated as AS201902), April 2020 (designated as AS202004), October 2020 (designated as AS202010R), and November 2020 (designated as AS202011). The collected sludge samples were immediately incubated to change the relative abundance of *Patescibacteria*. The AS202004 sample was anaerobically incubated for 3‍ ‍d and was then designated as AA202004. The AS202010R sample was aerobically incubated for 3‍ ‍d with washing and designated as AS202010A and without washing as AS202010B. In detail, 100‍ ‍mL of the AS202004 sample was transferred into a 120-mL sterilized vial, which was sealed with a butyl rubber stopper. The gas phase was replaced with nitrogen gas, and the vial was then incubated anaerobically at 20°C for 3 d. One hundred milliliters of activated sludge from sample AS202010R was washed with Elix water (Merck) and then incubated at 20°C for 3 d. In the present study, the samples AS201902, AS202004, and AA202004 were used in a metagenomic ana­lysis, while all seven samples were subjected to an amplicon ana­lysis. Fresh and incubated sludge samples were stored at –18°C for further ana­lyses.

### Amplicon ana­lysis of the 16S rRNA gene

DNA was extracted from activated sludge samples (0.5‍ ‍g wet weight) (AS201902, AS202004, AA202004, AS202010A, AS202010B, AS202010, and AS202011) using a FastDNA SPIN kit for soil (MP Biomedicals). PCR amplification was performed using a primer set for the V3–V4 region of the 16S rRNA genes (341F and 805R). The primer sequences, detailed PCR conditions, and purification procedures used are as previously described (Dinh *et al.*, 2021). Purified DNA was sequenced using a MiSeq platform with paired-end sequencing (2×300 bp) and a MiSeq Reagent kit (v.3; Illumina). The obtained sequences were trimmed, merged, clustered, and analyzed using QIIME 2 core 2021.11, as previously described (Bolyen *et al.*, 2019; Awata *et al.*, 2021; Kambara *et al.*, 2022). The SILVA 138 database (Quast *et al.*, 2013) was used for the assignment. To elucidate the relationship between *Patescibacteria* and other co-existing bacteria, operational taxonomic units (OTUs) that showed a relative abundance of >0.1% were extracted, and Spearman’s rank-order correlation coefficient was calculated for each OTU using Past 4.10 (Hammer *et al.*, 2001). OTUs that met the 5% significance level and correlated with *Patescibacteria* were investigated.

### Metagenomic ana­lysis

DNA was extracted from activated sludge samples (0.5‍ ‍g wet weight) (AS201902, AS202004, and AA202004) using a FastDNA SPIN kit for soil (MP Biomedicals). Extracted DNA was purified using Agencourt AMPure XP magnetic beads (Beckman Coulter Life Sciences). Illumina sequencing libraries were prepared for the three samples using a TruSeq DNA PCR Free (350) kit (Illumina) and paired-end sequenced (2×151 bp) using shotgun sequencing on a HiSeq X system (Illumina). PacBio sequencing libraries were prepared for three samples using a 20‍ ‍kb SMRTbell Express Template Prep kit (Pacific Biosciences of California) and sequenced on a PacBio Sequel II System (Pacific Biosciences of California). Circular consensus sequence (CCS) reads were generated from Sequel data with a Phred quality score above 20 (Q20, 99%).

A metagenomic ana­lysis was conducted as previously described (Hosokawa *et al.*, 2021). Raw paired-end reads from HiSeq X were trimmed using Trimmomatic v.0.39 (Bolger *et al.*, 2014). The trimmed reads from HiSeq X and CCS reads from PacBio Sequel II were co-assembled using SPAdes v.3.13.1 (Bankevich *et al.*, 2012). BBtools v38.84 was used to obtain mapping information. Contigs from the assembly were binned using MetaBAT2.0 (Kang *et al.*, 2019). The relative abundance of the bins (multi-contigs classified into a taxonomic microorganism) were calculated based on information from the mapping file (*i.e.*, coverage) generated in MetaBAT2.0. The completeness and contamination of the bins were assessed using CheckM v1.1.2 (Parks *et al.*, 2015). The 43 marker genes proposed by Brown *et al.* (2015) likely provide improved estimates of CPR genome quality. Contamination in the obtained bins was manually removed. The bins with contamination removed were annotated using Prokka v1.13 (Seemann, 2014) and DRAM v1.2.2 (Shaffer *et al.*, 2020). Predicted amino acid sequences were annotated using the KEGG BlastKEGG Orthology And Links Annotation (BlastKOALA) (Kanehisa *et al.*, 2016) and KEGG Automatic Annotation Server (KAAS) (Moriya *et al.*, 2007). BlastKOALA was used to visualize this pathway. A heatmap was created using KEGG-Decoder (Graham *et al.*, 2018) to visualize the percentage of gene possession related to each gene set. A phylogenetic tree of *Patescibacteria*, based on 400 marker protein sequences, was constructed using Phylophlan 3.0 (Asnicar *et al.*, 2020). The reference genome was selected from the genome registered in GenBank, and the complete genome was derived from activated sludge (Singleton *et al.*, 2021). Polyhydroxybutyrate (PHB) depolymerase-related sequences were aligned using mafft-linsi v7.480 (default parameters) (Katoh and Standley, 2013). Reference protein sequences were obtained from the top 500 hits for the identified PHB depolymerase-related protein (FNKGEGDK_00198) and known patescibacterial PHB depolymerase (OWK27304.1) using the NCBI-nr database. Protein sequences were clustered based on ≥70% similarity using CD-HIT version 4.8.1. (Fu *et al.*, 2012). A phylogenetic tree of PHB depolymerase-related proteins was constructed using iqtree2 version 2.1.2, with an automatically optimized substitution model of WAG+R10 (Minh *et al.*, 2020).

### Nucleotide sequence accession number

The sequence data of the partial 16S rRNA gene sequence were deposited in the GenBank/EMBL/DDBJ databases under the accession number DRA013509. Metagenomic sequence data were deposited in the DDBJ database under the DDBJ/EMBL/GenBank accession number DRA013531.

## Results and Discussion

### Amplicon ana­lysis of 16S rRNA genes

**Fig. 1.**
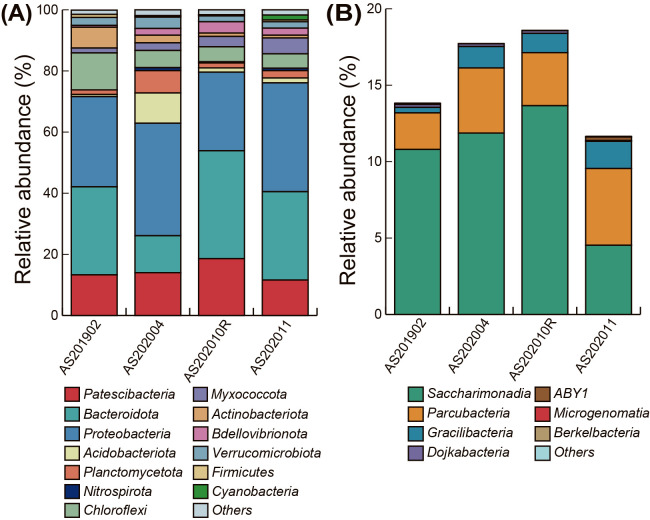
Microbial community composition at the phylum level in four activated sludge samples examined in the present study (A), and the detailed composition of *Patescibacteria* in four activated sludge samples (B) based on 16S rRNA gene amplicon sequencing. The total relative abundance of each sample in panel (B) corresponds to the relative abundance of *Patescibacteria* (red) in each sample in panel (A).

Amplicon sequencing of the 16S rRNA genes was performed to investigate the relative abundance of *Patescibacteria* in the seven activated sludge samples. On average, 34,573 reads and 549 OTUs were obtained from the seven samples (Table S3). In all activated sludge samples, except AS202004, *Patescibacteria* were predominant after *Proteobacteria* and *Bacteroidota*, with an average abundance of 12.1% (Fig. 1A). The most dominant group within *Patescibacteria* was *Saccharimonadia* in all samples, with the highest abundance of 13.7% in AS202010R. *Parcubacteria* and *Gracilibacteria* were the second and third most abundant groups, respectively (Fig. 1B). In addition to the above-mentioned groups, *Microgenomatia* (former candidate division OP11), ABY1, *Dojkabacteria* (former candidate division WS6), and *Berkelbacteria* were detected; however, their relative abundance was less than 0.2%. The ranges of the relative abundance of *Saccharimonadia*, *Parcubacteria*, and *Gracilibacteria* in untreated activated sludge samples (AS201902, AS202004, AS202010R, and AS202011) were 4.5–13.7%, 2.4–5.0%, and 0.4–1.8%, respectively. The relative abundance of *Patescibacteria* in all three treated samples (*i.e.*, aerobic and anaerobic incubations) decreased (Fig. S1). The relative abundance of *Saccharimonadia*, *Parcubacteria*, and *Gracilibacteria* in AS202004 were 9.5, 3.5, and 1.1%, respectively, whereas those in AA202004 were 5.5, 2.8, and 0.6%, respectively. The relative abundance of *Saccharimonadia*, *Parcubacteria*, and *Gracilibacteria* in AS202010R, AS202010A, and AS202010B were 9.5, 3.5, and 1.1%, 5.5, 2.8, and 0.6%, and 5.5, 2.8, and 0.6%, respectively. This decrease may be associated with the oxygen level or abundance of coexisting bacteria. The relative abundance of *Patescibacteria* in groundwater samples ranged between 2.1 and 20.7%; however, it was not possible to compare these samples directly because they were enriched using a filter-based sampling method (Danczak *et al.*, 2017).

### Correlation ana­lysis

**Fig. 2.**
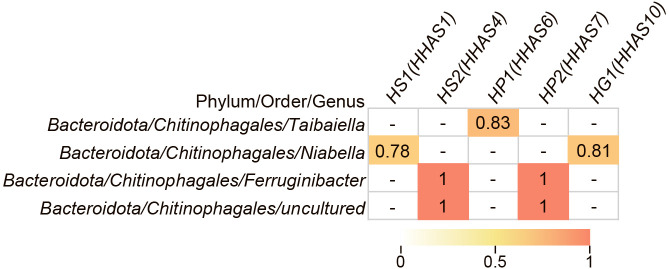
Spearman’s correlation between *Patescibacteria* and *Chitinophagales*. Correlation coefficients that met the 5% significance level are shown, and hyphens indicate that correlation coefficients did not meet the significance level. HS1 and HS2, HP1 and HP2, and HG1 indicate the OTUs of *Saccharimonadia*, *Parcubacteria*, and *Gracilibacteria*, respectively. The only OTUs belonging to *Patescibacteria* and *Bacteroidota* that matched with the reconstructed bins in the metagenomic ana­lysis with 100% sequence identity are shown. Correlations for all OTUs are shown in Fig. S1. Correlation coefficients that met the 5% significance level are presented as a heatmap. The parentheses indicate the bin ID shown in Table 1 with 100% sequence identity to the OTU.

Pearson’s correlation coefficients were calculated between the patescibacterial OTUs obtained from amplicon sequencing and other bacterial OTUs with more that 0.1% relative abundance. The OTUs of *Proteobacteria*, *Chloroflexi*, and *Planctomycetota* showed a positive correlation with the OTUs of *Patescibacteria*; however, in some cases, correlations were negative (data not shown). In contrast, the correlation between *Patescibacteria* and *Chitinophagales* belonging to the phylum *Bacteroidota* was positive (Fig. S2). Among the OTUs shown in Fig. S2, we extracted OTUs with a sequence that matched the reconstructed bin with 100% sequence identity (Fig. 2). Most *Chitinophagales* OTUs correlated with several patescibacterial OTUs (HS1, HS2, and HP2). In addition, two *Chitinophagales* OTUs correlated with the three lineages of *Patescibacteria*. A similar positive correlation between *Saccharimonadia* and *Chitinophagaceae* was found in acid mine drainage samples (Lemos *et al.*, 2019). Metabolic interactions between *Patescibacteria* and *Chitinophagales* are discussed in the following section.

### Genome reconstruction and basic information on bins

In total, 0.77 billion reads and 0.06 million reads were obtained from HiSeq X and PacBio CCS sequencing of the three activated sludge samples, respectively (Table S2). The hybrid assembly using HiSeq X and PacBio CCS reads generated 12,097 contigs with an N50 value of 148,787 bp. A total of 8,211 contigs >1,500 bp were extracted and classified into 320 bins. Ten patescibacterial bins were reconstructed, which consisted of *Saccharimonadia* (five bins, HHAS1–HHAS5), *Parcubacteria* (four bins, HHAS6–HHAS9), and *Gracilibacteria* (one bin, HHAS10) (Table 1). The completeness of *Sacchrimonadia* and *Parcubacteria* ranged between 88.4 and 97.7 and between 62.8 and 90.7%, respectively, while that of *Gracilibacteria* was 97.7%.

**Fig. 3.**
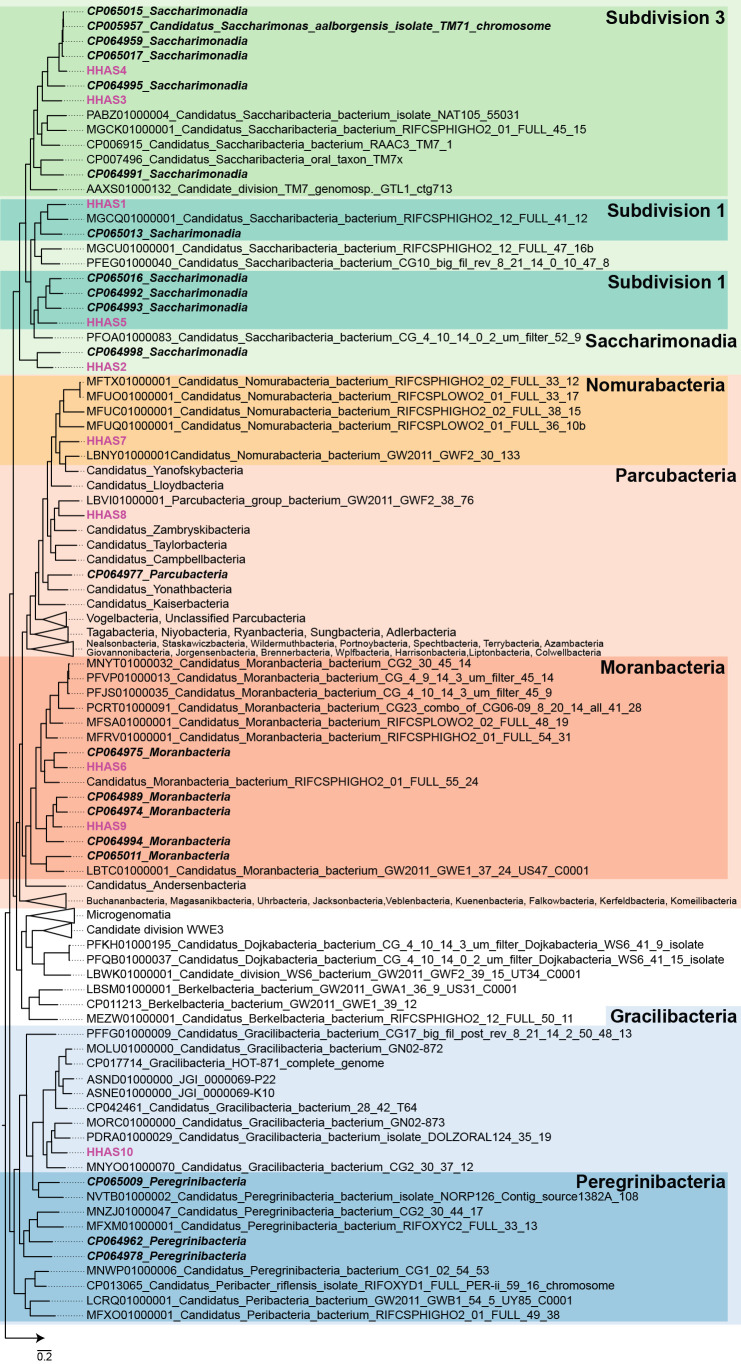
Genome-based phylogenetic tree of reconstructed patescibacterial bins in activated sludge and related genomes. The bins found in the present study are shown in pink. Phylum-level designations (dotted line) are highlighted on the right. Bold italics indicate genomes obtained from activated sludge samples.

A phylogenetic tree of the ten bins based on the protein sequence is shown in Fig. 3. Bins belonging to *Saccharimonadia* were classified into three groups. The group including HHAS3 and HHAS4 was related to the well-described saccharimonadial species *Candidatus* Saccharimonas aalborgensis (CP005957), reconstructed from a Danish activated sludge sample (Albertsen *et al.*, 2013). Since this species shows a small coccus morphology (Albertsen *et al.*, 2013), HHAS3 and HHAS4 were also considered to be small cocci. The HHAS1 and HHAS5 groups were related to the genomes of activated sludge samples. According to sequence similarities based on 16S rRNA genes, the morphology of this group was primarily filamentous (Kindaichi *et al.*, 2016). The morphology of filamentous *Saccharimonadia* needs to be confirmed using FISH in the future. The HHAS2 bin formed a different clade from other saccharimonadial genomes with a genome from activated sludge (Singleton *et al.*, 2021). However, the details of this group remain largely unknown. The parcubacterial bins were classified into three groups. The HHAS7 bin was classified as *Nomurabacteria* and was related to genomes from groundwater samples (Brown *et al.*, 2015). Groups HHAS6 and HHAS9 belonged to *Moranbacteria*. In addition, clades HHAS6 and HHAS9 consisted of genomes from activated sludge samples (Singleton *et al.*, 2021). The details of *Moranbacteria* in activated sludge samples are also unclear because the majority of information on *Moranbacteria* was obtained from groundwater samples (Anantharaman *et al.*, 2016). The HHAS8 bin did not belong to any parcubacterial subgroup. HHAS10 was classified as belonging to *Gracilibacteria*. Although some of the gracilibacterial genomes were also reconstructed from activated sludge samples (Singleton *et al.*, 2021), the HHAS10 bin formed a clade that included genomes from human oral samples (Dudek *et al.*, 2017).

### Metabolic ana­lysis

**Fig. 4.**
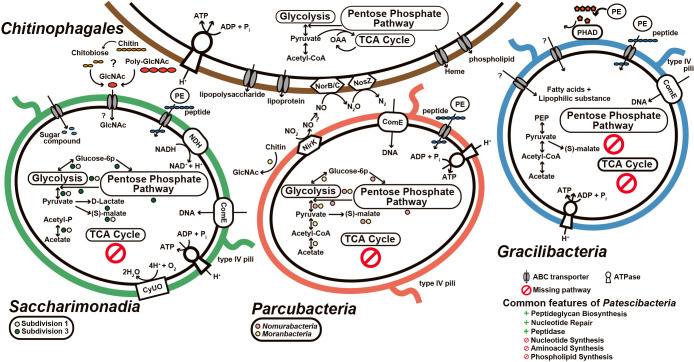
Predicted metabolic potential of *Gracilibacteria*, *Parcubacteria*, and *Saccharimonadia*, and putative metabolic interactions between *Patescibacteria* and *Chitinophagales* based on the genetic information obtained from the metagenomic ana­lysis. The colored circles below *Saccharimonadia* (light green and green) and *Parcubacteria* (pink and yellow) indicate the presence of genes only found in the subdivision/subgroup members. Semicircles indicate that one of the two possessed the genes. The common features of *Patescibacteria* are also shown. Abbreviations: CyUO, cytochrome O ubiquinol oxidase; NDH, NADH dehydrogenase; PHAD, polyhydroxyalkanoate depolymerase; ComEC, competence protein ComEC; PE, peptidases; OAA, oxaloacetate; GlcNAc, N-acetylglucosamine; PEP, phosphoenolpyruvate.

The predicted metabolic potential of *Gracilibacteria*, *Parcubacteria*, and *Saccharimonadia*, and the putative metabolic interactions between *Patescibacteria* and *Chitinophagales* based on the metagenomic ana­lysis in this study are shown in Fig. 4. Patescibacterial bins revealed that *Patescibacteria* did not possess *de novo* nucleotide synthesis, amino acid synthesis, phospholipid synthesis, or a full TCA cycle. In addition, *Patescibacteria* possessed ABC transporters with unknown functions, the peptidoglycan biosynthesis pathway, and type IV pili (Fig. S3). The lack of *de novo* amino acid synthesis suggests that peptidases acquire amino acids. The presence of peptidases was also confirmed (Table S4). Several patescibacterial bins converted glycine to serine and harbored serine peptidases. These common features are consistent with the genomes of activated sludge samples as well as other natural samples (Wrighton *et al.*, 2012, 2014; Albertsen *et al.*, 2013; Danczak *et al.*, 2017; Starr *et al.*, 2018; Lemos *et al.*, 2019; 2020; Sieber *et al.*, 2019; Chaudhari *et al.*, 2021; Moreira *et al.*, 2021; Yakimov *et al.*, 2021). The incomplete nucleotide synthesis pathway and the presence of the *com*E gene and type IV pili support the acquisition of DNA from outside cells (Chen and Gotschlich, 2001; Starr *et al.*, 2018).

Saccharimonadial bins possessed glycolysis and the pentose phosphate pathway, with possession patterns depending on the subgroup (Fig. 4). The members of subdivision 1 (HHAS1 and HHAS5), which are putative filamentous *Saccharimonadia*, partially possessed glycolysis, whereas members of subdivision 3 (HHAS3 and HHAS4) possessed both glycolysis and the pentose phosphate pathway. The possession of genes to convert pyruvate to lactate, acetate, and malate and the lack of a TCA cycle supports fermentative metabolism. These results are consistent with previous findings (Wrighton *et al.*, 2012, 2014; Albertsen *et al.*, 2013; Danczak *et al.*, 2017; Starr *et al.*, 2018; Lemos *et al.*, 2019; 2020; Sieber *et al.*, 2019; Moreira *et al.*, 2021; Yakimov *et al.*, 2021). The fermentative pathway from pyruvate to lactate or malate may facilitate the production of NAD^+^ (Starr *et al.*, 2018; Lemos *et al.*, 2019). The pentose phosphate pathway in subdivision 3 may be involved in the conversion of glucose-6P to glyceradehyde-3P and in energy conversion (NADPH to NADP production) (Albertsen *et al.*, 2013). However, genes involved in the synthesis of nucleic acids, such as ribose-phosphate pyrophosphokinase, were absent. Therefore, they are not expected to contribute to anabolism (the production of deoxyribonucleotides) (Castelle *et al.*, 2018). All reconstructed saccharimonadial bins in the present study possessed the NADH dehydrogenase-like protein and complete cytochrome o ubiquinol oxidase, which is related to the oxygen scavenging system, despite the absence of the TCA cycle (Kantor *et al.*, 2013; Starr *et al.*, 2018; Lemos *et al.*, 2019). In addition, Lemos *et al.* (2019) suggested that *Saccharimonadia* follow non-obligatory fermentative metabolism with occasional aerobic respiration. As previously reported by Lemos *et al.* (2019), *Saccharimonadia* have membrane-bound NADH dehydrogenase to supply NAD^+^ and pass the electron to ubiquinone, which transfers it to cytochrome O ubiquinol oxidase. Cytochrome then reduces O_2_ to H_2_O as the final receptor, delivering protons through the plasma membrane to generate the proton electromotive force used for ATP synthesis by ATP synthase. Filamentous *Saccharimonadia* in activated sludge took up N-acetylglucosamine under aerobic conditions, as demonstrated by microautoradiography combined with FISH (Kindaichi *et al.*, 2016). *Chitinophagales* bins (HHAS12, HHAS13, and HHAS14) possessed chitinase (MHHEHLFG_00073, MHHEHLFG_01825, AECFEMFL_01184, and KHEBLPDM_00108) and all *Chitinophagales* bins harbored beta-acetylhexosamidase (MHHEHLFG_00810, CLEEFKKN_01010, AECFEMFL_02009, and KHEBLPDM_01401). *Chitinophagales* have the potential to convert chitin to N-acetylglucosamine via chitobiose (Fig. 4). All *Chitinophagales* bins encoded poly-beta-1,6 N-acetyl-d-glucosamine synthase (PgaC). This enzyme catalyzes the polymerization of uridine diphosphate-N-acetylglucosamine to produce poly-N-acetylglucosamine (PGA). There were other bins in active sludge belonging to *Ignavibacteria*, *Acidobacteria*, *Actinobacteria*, *Bacteroidota*, *Chloroflexi*, *Nitrospira*, *Proteobacteria*, and *Verrucomicrobia*. These bacteria possessed PgaC and were present in approximately 38–41% of samples from AS201902, AS202004, and AA202004. Filamentous *Saccharimonadia* in activated sludge took up N-acetylglucosamine, which strongly supports the metabolic interaction between *Chitinophagales* and *Saccharimonadia* via N-acetylglucosamine in activated sludge. In addition, *Saccharimonadia* have been suggested to use some of the PGA produced by bacteria (Hosokawa *et al.*, 2021). The mechanisms by which N-acetylglucosamine is assimilated or catabolized by *Saccharimonadia* currently remain unclear. Investigations on the metabolism of incorporated N-acetylglucosamine are highly challenging, but are warranted.

Parcubacterial bins possessed glycolysis and/or the pentose phosphate pathway. Parcubacterial bins also encoded genes involved in the conversion of pyruvate to acetate and malate (Fig. 4). The possession of these pathways and the lack of a TCA cycle are similar features to those of saccharimonadial bins and support fermentative metabolism, as reported in previous studies (Wrighton *et al.*, 2012; 2014; Albertsen *et al.*, 2013; Danczak *et al.*, 2017; Lemos *et al.*, 2019; 2020; Starr *et al.*, 2018; Sieber *et al.*, 2019; Moreira *et al.*, 2021; Yakimov *et al.*, 2021). A moranbacterial bin (HHAS9) possessed chitinase (DEAMOMGP_00832 and DEAMOMGP_00955) (Fig. 4), but not the genes to convert N-acetylglucosamine to other compounds. The nomurabacterial bin (HHAS7) possessed the copper-containing nitrite reductase gene (JCNBLHJH_00228, *nirK*) (Fig. 4). Some members of *Parcubacteria* are known to be involved in nitrite reduction (Castelle *et al.*, 2017; Danczak *et al.*, 2017; He *et al.*, 2021). In addition, several *Chitinophagales* bins (HHAS12 and HHAS13) possessed the nitric oxide reductase subunit B/C (*norB/C*) (CLEEFKKN_01663, CLEEFKKN_01664, AECFEMFL_01795, and AECFEMFL_01795) and nitrous-oxide reductase (*nosZ*) (CLEEFKKN_01654 and AECFEMFL_02605). Therefore, *Nomurabacteria* were partially responsible for denitrification along with *Chitinophagaceae* in the activated sludge process.

The gracilibacterial bin (HHAS10) had negligible central carbon metabolism. It possessed only pyruvate kinase (FNKGEGDK_00842), malate dehydrogenase (FNKGEGDK_00357), and 2-oxoglutarate/2-oxoacid ferredoxin oxidoreductase (FNKGEGDK_00164 and FNKGEGDK_00166). Although the poor metabolic potential of *Gracilibacteria* has also been reported (Sieber *et al.*, 2019), the gracilibacterial genomes in previous studies were mainly reconstructed from other habitats, such as oral and ground water samples. The genome size of the HHAS10 bin was 1.3‍ ‍Mbp, which is similar to that of other *Gracilibacteria* (Sieber *et al.*, 2019), and completeness was relatively high (Table 1). Nevertheless, it was not possible to predict the metabolic potential of *Gracilibacteria* reconstructed in the present study using the current databases. The accumulation of genomic information on *Gracilibacteria* in activated sludge is necessary to construct substantial databases. In contrast, the gracilibacterial bin (HHAS10) possessed four copies of peptidase belonging to the M23 family (Table S4), which lyses the cell walls of other microorganisms. The HHAS10 bin also possessed a phospholipase gene (FNKGEGDK_00603, FNKGEGDK_00841), whereas *Chitinophagales* possessed an ABC transporter (MHHEHLFG_00269, MHHEHLFG_00294, MHHEHLFG_00428, MHHEHLFG_01772, MHHEHLFG_01773, MHHEHLFG_01879, KHEBLPDM_00308, KHEBLPDM_00309, and KHEBLPDM_02272), which releases phospholipids (Fig. 4). Therefore, the metabolic flow of phospholipids between *Chitinophagales* and *Gracilibacteria* in activated sludge was considerable. The HHAS10 bin possessed a homolog of polyhydroxyalkanoate (PHA) depolymerase (FNKGEGDK_00792), which showed >40% homology (<1e-65) to PHA depolymerases of known species (Table 2). This feature may help to obtain an energy source, even though *Gracilibacteria* have negligible central carbon metabolism. In general, PHA is degraded by PHA depolymerase to monomers, such as 3HB, which are then oxidized to acetoacetyl-CoA in a reaction catalyzed by 3HB dehydrogenase. This is then converted to acetyl-CoA by β-ketothiolase (Ong *et al.*, 2017). Although *Candidatus* Parcunitrobacter nitroensis belonging to *Parcubacteria* also possessed PHB, which is a PHA, depolymerase, and peptidase that acts extracellularly and converts PHB to acetate, suggesting that PHB may be used as a carbon source (Castelle *et al.*, 2017), no genes related to the reaction pathway of hydroxybutyrate in the HHAS10 bin were identified. Based on an amino acid sequence homology search using the NCBI-nr database, we found that the genome of HHAS10 bin had a surface layer protein (FNKGEGDK_00198) that was widely conserved in gracilibacterial genomes with high similarity (Table 2 and Fig. S4). Besides, the proteins showed 28% (41/145 bp, 3e-15) and 27–30% (<1e-16) homology with the PHB depolymerases of *Candidatus* Parcunitrobacter nitroensis (OWK27304.1) and other taxa (*Myxococcales*, *Sorangium cellulosum*, and *Streptomyces* sp.), respectively. Further studies are needed on the generality and roles of PHA/PHB depolymerases in *Gracilibacteria*.

## Conclusions

In the present study, the metabolic potential of *Patescibacteria* was predicted from the MAGs of activated sludge samples, and the physiological role of *Patescibacteria* in activated sludge was estimated. The genomes of three *Saccharimonadia*, three *Parcubacteria*, and one *Gracilibacteria* species revealed a lack of *de novo* nucleotide synthesis, amino acid synthesis, phospholipid synthesis, and a full TCA cycle. Ten reconstructed genomes showed a strong positive correlation of relative abundance with *Chitinophagales* based on 16S rRNA genes. Metabolic interactions between a member of *Saccharimonadia* and *Chitinophagales* via N-acetylglucosamine, between a member of *Parcubacteria* and *Chitinophagales* via nitrogen compounds related to denitrification, and between *Gracilibacteria* and *Chitinophagales* via phospholipids in activated sludge were supported by metabolic predictions from 10 recovered *Patescibacteria* MAGs and five *Chitinophagales* MAGs. The high abundance of peptidases in *Gracilibacteria* suggests their role in cell lysis in activated sludge. Further studies related to visualization with FISH and the enrichment of *Patescibacteria* are necessary to elucidate the *in situ* physiological roles of *Patescibacteria* in the activated sludge process.

## Citation

Fujii, N., Kuroda, K., Narihiro, T., Aoi, Y., Ozaki, N., Ohashi, A., and Kindaichi, T. (2022) Metabolic Potential of the Superphylum *Patescibacteria* Reconstructed from Activated Sludge Samples from a Municipal Wastewater Treatment Plant. *Microbes Environ ***37**: ME22012.

https://doi.org/10.1264/jsme2.ME22012

## Supplementary Material

Supplementary Material

## Figures and Tables

**Table 1.** Characteristics of patescibacterial bins obtained in the present study

**Bin ID**	**Taxonomy**	**Bin size (Mbp)**	**Completeness (%)**	**Contamination (%)**	**Number of contigs**	**Number of CDSs**	**Relative abundance (%)** ^†^		
							**AS201902**	**AS202004**	**AA202004**
HHAS1	*Saccharimonadia*	0.91	88.37*	0*	3	946	1.79	0.04	0.03
HHAS2	*Saccharimonadia*	0.83	97.67*	0*	3	847	0.20	0	0
HHAS3	*Saccharimonadia*	1.00	93.02*	0*	2	1027	1.47	0	0
HHAS4	*Saccharimonadia*	0.73	90.70*	0*	3	759	2.26	0	0
HHAS5	*Saccharimonadia*	0.71	88.37*	0*	6	746	0.29	0	0
HHAS6	*Parcubacteria*	0.53	90.70*	0*	8	536	0.15	0	0
HHAS7	*Parcubacteria*	0.60	62.79*	0*	2	627	0.48	0	0
HHAS8	*Parcubacteria*	0.60	90.70*	0*	5	619	0.02	0.26	0.19
HHAS9	*Parcubacteria*	0.96	79.07*	0*	6	942	0.29	0.27	0.44
HHAS10	*Gracilibacteria*	1.30	97.67*	0*	3	1175	0.02	0.22	0.10
HHAS11	*Chitinophagales*	2.37	75.24	0	17	2051	0	0.17	0.23
HHAS12	*Chitinophagales*	2.79	75.2	3.96	21	2435	0.97	0	0.02
HHAS13	*Chitinophagales*	3.13	94.77	3.45	5	2669	1.85	0	0
HHAS14	*Chitinophagales*	2.99	91.21	2.72	14	2354	0.78	0	0

* Calculated using the CPR marker set. ^†^ Calculated based on the mapping file generated in MetaBAT2.0

**Table 2.** Summary of genes related to polyhydroxyalkanoate and polyhydroxybutyrate depolymerases in the *Gracilibacteria* bin HHAS10

**Locus tags**	**Amino acid identity to known proteins of representative taxonomies based on the NCBI-nr database**			
	**Description**	**Accession no.**	**Identities**	**e-value**
FNKGEGDK_00792	polyhydroxyalkanoate depolymerase [*Alteromonas oceani*]	WP_123327345.1	138/317 (44%)	6e-68
	polyhydroxyalkanoate depolymerase [*Polynucleobacter paneuropaeus*]	WP_215313699.1	144/340 (42%)	7e-68
	polyhydroxyalkanoate depolymerase [*Alteromonas lipolytica*]	WP_070177576.1	137/317 (43%)	2e-67
	polyhydroxyalkanoate depolymerase [*Polynucleobacter wuianus*]	WP_216235107.1	144/340 (42%)	2e-67
	polyhydroxyalkanoate depolymerase [*Marisediminitalea aggregata*]	WP_073324272.1	137/317 (43%)	4e-66
FNKGEGDK_00198	hypothetical protein US76_00085 [*Parcubacteria* GW2011_GWA2_38_13b]	OWK27304.1	41/145 (28%)	3e-15
	S-layer homology domain-containing protein [*Candidatus* Gracilibacteria bacterium]	MBP9812078.1	305/443 (69%)	0
	S-layer homology domain-containing protein [*Candidatus* Gracilibacteria bacterium]	MBC7498061.1	267/427 (63%)	0
	Ricin and poly(3-hydroxybutyrate) depolymerase fusion [*Myxococcales* bacterium]	MCA9656682.1	64/240 (27%)	9e-22
	Ricin and poly(3-hydroxybutyrate) depolymerase fusion [*Sorangium cellulosum*]	KYF79897.1	65/225 (29%)	4e-19
	poly(3-hydroxybutyrate) depolymerase [*Streptomyces* sp. TLI_55]	SNX55894.1	87/292 (30%)	4e-17
