# Supplementary material for: Metabolic Potential of the Superphylum Patescibacteria Reconstructed from Activated Sludge Samples from a Municipal Wastewater Treatment Plant
Source: Microbes Environ. 2022 Jun 28;37(3):ME22012. doi: 10.1264/jsme2.ME22012 (PMC9530719; doi:10.1264/jsme2.ME22012)
Supplement: Supplementary file 1 — Supplementary Material [file 37_22012_s1.pdf]

Metabolic potential of the superphylum *Patescibacteria* reconstructed from activated sludge samples from a municipal wastewater treatment plant

Naoki Fujii[1], Kyohei Kuroda[2], Takashi Narihiro[2], Yoshiteru Aoi[3], Noriatsu Ozaki[1], Akiyoshi Ohashi[1], Tomonori Kindaichi[1*]

Table S1. Physicochemical characteristics of influent wastewater in the wastewater treatment plant

Parameter	Range	Average ± Standard deviation (n = 5)
Yearly-averaged wastewater temperature (°C)	23.2–24.1	23.8 ± 0.3
pH	6.9–7.2	7.0 ± 0.1
BOD_5	139.5–209.0	180.7 ± 27.3
COD	90.6–127.0	102.1 ± 13.0
SS (mg/L)	125.9–168.2	147.4 ± 16.6
T-N	40.0–61.0	48.5 ± 7.5
T-P	2.12–3.87	3.3 ± 0.6

Table S2. Summary of metagenomic data used in this study

Platform	Sample	Total read bases (bp)	Total reads	GC (%)	Q20 (%)
HiSeq X	AS201902	41,297,169,086	273,491,186	56.2	95.22
HiSeq X	AS202004	37,050,204,090	245,365,590	62.5	94.80
HiSeq X	AA202004	37,356,130,392	247,391,592	61.9	94.40
PacBio	AS201902	504,086,620	41,445	50.6	100
PacBio	AS202004	152,307,751	16,176	57.3	100
PacBio	AA202004	10,256,530	1,426	55.0	100

Table S3. Summary of amplicon sequence data used in this study

Sample Name	Number of paired reads	Number of nonchimeric paired reads	Number of OTUs
AS201902	70,110	37,331	550
AS202004	83,355	47,205	659
AA202004	88,682	35,925	581
AS202010A	48,203	26,927	462
AS202010B	56,035	32,116	507
AS202010R	45,071	26,817	475
AS202011	52,497	35,693	609

Table S4. Details of the peptidase possessed by *Patescibacteria*

gene_id	HHAS1	HHAS2	HHAS3	HHAS4	HHAS5	HHAS6	HHAS7	HHAS8	HHAS9	HHAS10
M41	1	1	1	1	2	1	1	1	0	2
M01	1	0	1	1	1	0	1	0	0	0
M17	0	0	0	0	0	0	0	0	1	1
M38	0	1	0	0	0	1	0	0	1	0
S66	0	0	0	1	0	0	0	0	0	0
C40	0	1	1	0	0	0	0	1	0	0
C60A	2	2	2	1	0	0	1	0	0	2
C39	0	0	0	1	0	0	0	0	1	0
S14	1	1	1	1	1	0	1	0	1	1
A08	0	0	0	0	0	0	0	0	0	1
M26	0	1	0	0	0	0	0	0	0	0
M82	0	2	0	0	0	0	1	0	0	0
S49C	0	0	0	0	0	0	0	0	1	0
U32	0	0	0	0	0	0	0	0	0	1
C51	0	0	0	0	0	1	0	0	0	0
M23B	1	1	1	1	0	1	1	2	1	4
S16	3	2	2	2	2	1	1	1	0	2
S33	0	1	1	1	1	0	0	0	0	0
S51	1	0	0	0	3	0	1	0	0	1
M24A	0	1	1	1	0	1	1	1	1	1
M24B	0	0	0	1	0	0	0	0	0	1
M20A	1	1	1	0	1	0	1	0	2	1
M20F	0	0	1	0	0	0	0	0	0	0
C82A	0	1	0	0	1	0	0	1	1	0
I63	0	1	0	0	0	0	0	0	0	0
I01	0	0	0	0	0	0	0	0	0	1
I87	0	1	2	1	0	0	1	1	0	1
I39	0	0	0	0	0	0	0	0	0	1
S54	0	0	0	0	0	0	0	0	0	1
A24A	1	1	1	1	2	0	1	1	0	1
M43B	0	1	1	0	0	1	0	0	1	0
M48A	1	0	0	1	0	0	0	1	0	0
M48B	0	1	1	0	0	1	1	0	1	2

Table S4. Continued

gene_id	HHAS1	HHAS2	HHAS3	HHAS4	HHAS5	HHAS6	HHAS7	HHAS8	HHAS9	HHAS10
M48C	0	0	0	0	0	0	0	0	0	1
M50B	2	2	1	2	2	1	2	1	1	1
M10A	0	0	0	0	0	0	0	0	1	0
M10B	0	0	0	1	0	0	0	0	0	0
M16A	0	0	0	1	0	0	0	0	0	0
M16B	0	0	0	1	0	1	0	0	0	0
M13	0	0	1	0	0	0	0	0	0	0
M03A	0	0	0	0	0	0	0	1	0	0
M03B	0	0	0	0	0	0	0	0	0	1
M15B	4	0	1	2	1	0	0	1	0	0
M15C	1	1	0	0	0	0	2	0	0	0
M15D	0	0	1	0	0	0	0	0	0	1
C26	3	3	1	1	1	1	1	2	2	4
S26A	1	1	2	1	2	1	1	1	0	1
S26B	0	0	0	0	1	0	0	0	0	0
S13	0	0	0	0	0	0	1	0	0	0
C56	0	0	0	0	0	0	1	0	0	0
S29	0	0	0	0	0	0	0	0	1	0
N11	1	1	1	1	1	1	0	1	2	1
U69	0	0	1	0	0	0	0	1	0	1
C44	1	0	1	1	0	0	0	0	1	0
S08A	1	1	1	1	0	0	1	0	0	0
S01A	0	0	0	0	0	0	0	0	0	1
S01B	0	0	1	0	0	0	0	0	0	0
S01C	1	1	1	1	1	0	0	0	0	1
S41A	1	1	1	1	1	1	1	1	1	1
S09D	0	0	0	0	0	0	1	1	0	0
S11	0	1	0	0	0	2	1	1	1	1
M79	1	1	2	3	1	0	0	1	0	2
S24	1	1	2	0	1	1	2	0	1	2
M95	2	0	1	1	0	0	0	1	0	1

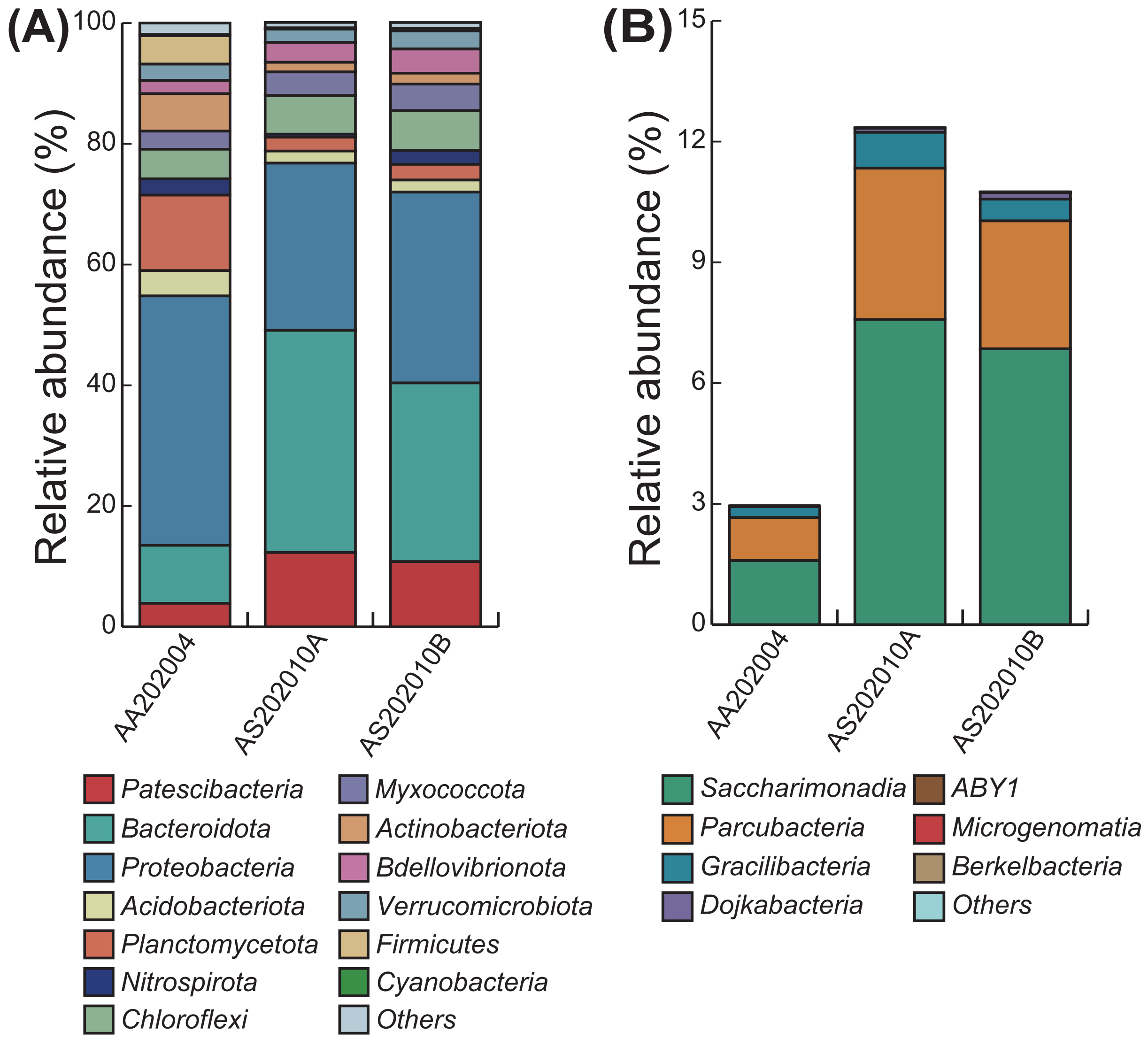

Fig. S1 Results of Amplicon sequencing analysis of treated activated sludge samples. Microbial community composition at the phylum level in the three treated activated sludge samples used in this study (A) and the detailed composition of *Patescibacteria* in the three treated activated sludge samples (B) based on 16S rRNA gene amplicon sequencing. The total relative abundance of each sample in panel (B) corresponds to the relative abundance of *Patescibacteria* (red) in each sample in panel (A).

Phylum/Order/Genus	HS1(HHAS1)	HS2(HHAS4)	HP1(HHAS6)	HP2(HHAS7)	HP3(HHAS9)	HG1(HHAS10)
Bacteroidota/Chitinophagales/37-13	-	-	-	-	-	-
Bacteroidota/Chitinophagales/Ferruginibacter	-	-	0.83	-	-	-
Bacteroidota/Chitinophagales/Ferruginibacter	-	-	0.87	-	-	0.76
Bacteroidota/Chitinophagales/Ferruginibacter	-	-	-	-	-	-
Bacteroidota/Chitinophagales/Ferruginibacter	-	1	-	1	-	-
Bacteroidota/Chitinophagales/Ferruginibacter	-	-	0.75	-	-	-
Bacteroidota/Chitinophagales/Ferruginibacter	0.93	-	-	-	-	0.90
Bacteroidota/Chitinophagales/Ferruginibacter	-	-	-	-	-	-
Bacteroidota/Chitinophagales/Niabella	0.78	-	-	-	-	0.81
Bacteroidota/Chitinophagales/Taibaiella	-	-	-	-	-	-
Bacteroidota/Chitinophagales/Taibaiella	-	-	0.83	-	-	-
Bacteroidota/Chitinophagales/Taibaiella	-	-	-	-	-	-
Bacteroidota/Chitinophagales/Terrimonas	-	-	-	-	-	-
Bacteroidota/Chitinophagales/Terrimonas	-	-	-	-	-	-
Bacteroidota/Chitinophagales/uncultured	-	-	-	-	-	-
Bacteroidota/Chitinophagales/uncultured	-	-	0.78	-	-	-
Bacteroidota/Chitinophagales/UTBCD1	-	-	0.86	-	-	-
Bacteroidota/Chitinophagales/OLB8	0.73	-	-	-	-	-
Bacteroidota/Chitinophagales/uncultured	-	1	-	1	-	-
Bacteroidota/Chitinophagales/uncultured	-	-	-	-	-	-
Bacteroidota/Chitinophagales/uncultured	-	-	-	-	-	-
Bacteroidota/Chitinophagales/uncultured	-0.81	-	-	-	0.79	-0.74
Bacteroidota/Chitinophagales/uncultured	-	-	-	-	-	-
Bacteroidota/Chitinophagales/uncultured	-	-	-	-	-	-
Bacteroidota/Chitinophagales/uncultured	0.81	-	-	-	-	0.74
Bacteroidota/Chitinophagales/uncultured	-	0.78	-	0.78	-	-
Bacteroidota/Chitinophagales/uncultured	-	-	-0.75	-	0.76	-
Bacteroidota/Chitinophagales/uncultured	-	-	0.87	-	-	0.76
Bacteroidota/Chitinophagales/uncultured	-	-	-	-	-	-
Bacteroidota/Chitinophagales/uncultured	-	-	0.81	-	-	-
Bacteroidota/Chitinophagales/uncultured	-	-	0.83	-	-	-
Bacteroidota/Chitinophagales/uncultured	-	-	0.80	-	-	-
Bacteroidota/Chitinophagales/uncultured	0.75	-	0.81	-	-	0.77

-1 -0.5 0 0.5 1

Fig. S2 Results of correlation analysis between *Patescibacteria* and *Chitinophagales*. Spearman's correlation between *Patescibacteria* and *Chitinophagales*. Correlation coefficients that met the 5% significance level are shown, and hyphens indicate that the correlation coefficients did not meet the significance level. HS1 and HS2, HP1, HP2 and HP3, and HG1 indicate the OTU of *Saccharimonadia*, *Parcubacteria*, and *Gracilibacteria*, respectively. Correlation coefficients that met the 5% significance level are presented as a heatmap. The parentheses indicate the bin ID shown in Table 1 with 100% sequence identity to the OTU.

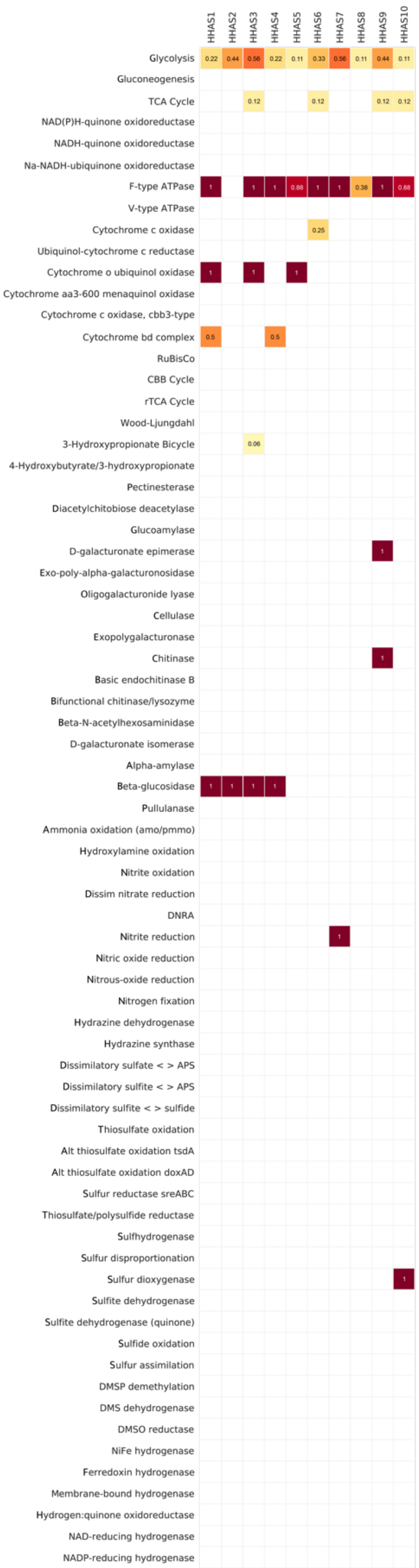

Fig. S3 Heatmap showing the metabolic function of patescibacterial bins based on KEGG and Blastp. The ID at the top of the heat map indicates the bin IDs in Table 1. The numbers in the boxes indicate the percentage of related genes possessed, with 1 indicating possession of the entire gene set. Similarly, the heat map shows the percentage of possession of related genes.

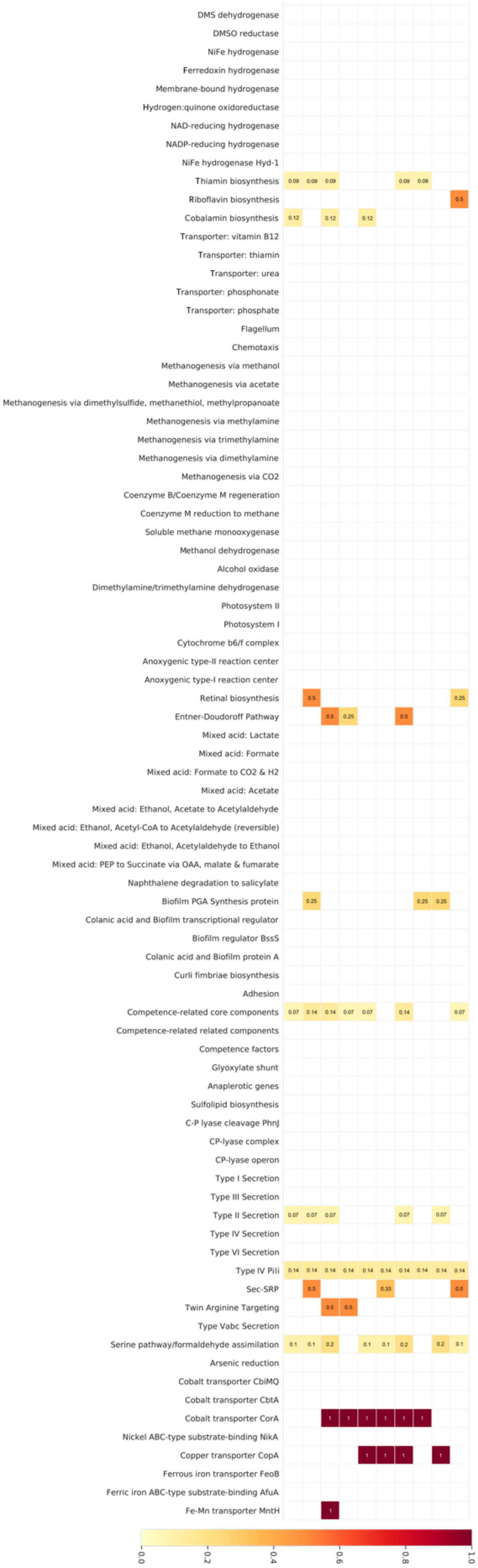

Fig. S3 Continued.

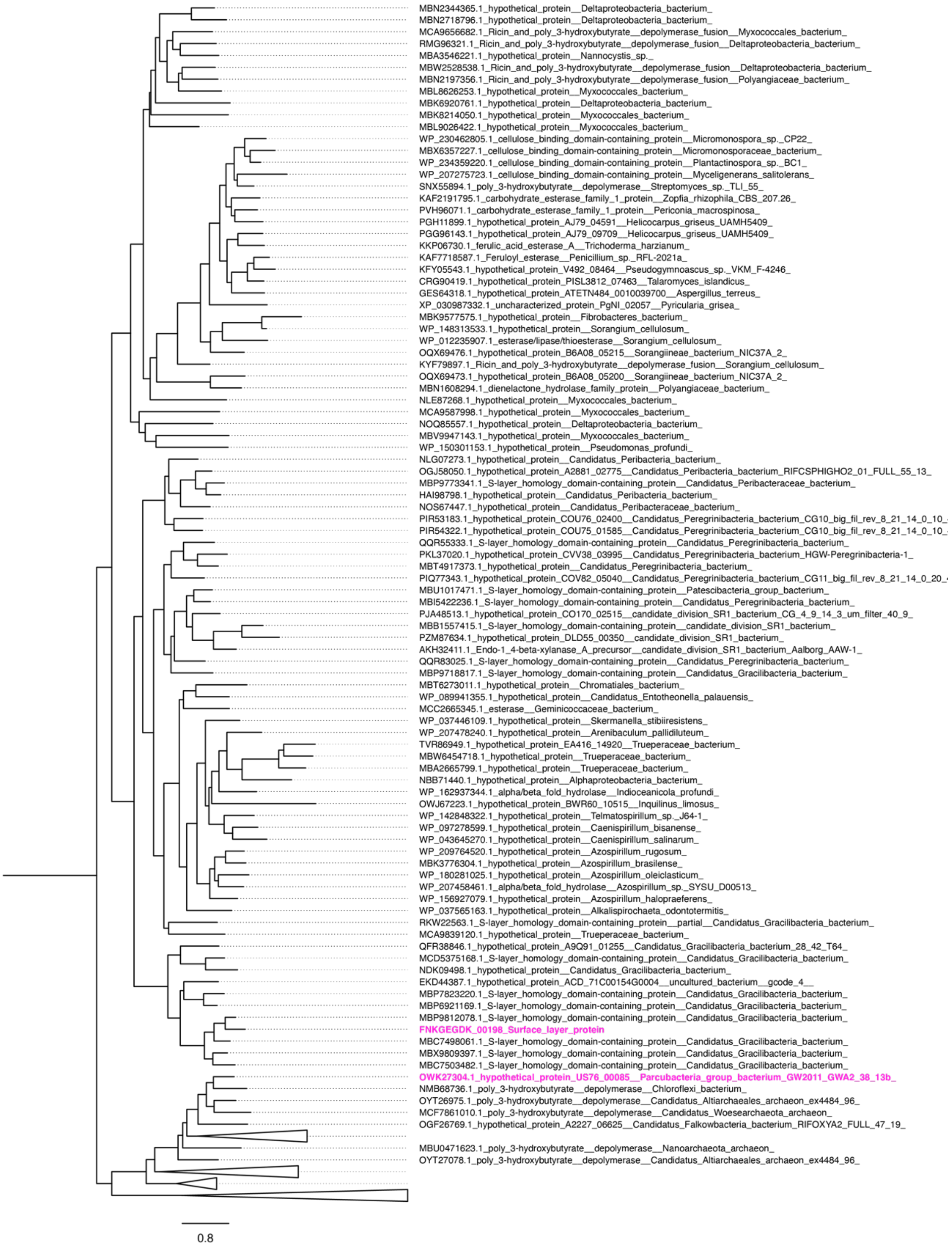

Fig. S4 Tree of PHB depolymerases.
